# The Hygiene of the Asthmatic1Read at the Buffalo Academy of Medicine, November 14, 1899.

**Published:** 1900-01

**Authors:** George N. Jack

**Affiliations:** Depew, N. Y.


### THE HYGIENE OF THE ASTHMATIC.[1]

By GEORGE N. JACK, M. D., Depew, N. Y.

LAST December I read a paper before this Academy, entitled Abnormalities of the blood—the etiology of asthma. That paper furnished ample proof that asthma is a disease due to an abnormal blood biochemy, which impairs its oxygenation; that the nerves and lungs take no part in its etiology, other than the performance of a physiological duty; and that the blood dyscrasias,

1. Read at the Buffalo Academy of Medicine, November 14, 1899.

either singly or collectively, that figure most prominently in its etiology are the anemic, the leukemic and the lithemic. It was understood that these terms were used only in a comparative sense for the want of a more definite phraseology, and they are named in the order of their importance. Or, in other words, it was shown that the abnormal blood biochemy that produced asthma was usually associated with anemia, less frequently with leukemia and occasionally with lithemia.

The object, then, of a special hygiene for the asthmatic is to encourage such a mode of life as will tend to procure and maintain a normal blood biochemy. This makes it an exceedingly interesting, delicate and difficult subject. Difficult, because there is no recognised literature on the subject. Interesting, because it has, in the hands of quacks and advertised physicians, contributed largely to the production of permanent cures of thousands of suffering asthmatics, while the regular profession has been compelled to dispute the cures, or behold them with bewildered amazement. Delicate, because it deals with a most accurate, sensitive, definite and automatic, chemical and electrical phenomenon, a chemistry of life that beguiles imagination, defies demonstration and discourages imitation.

It is not the intention of this paper to mystify, theorise, nor idly speculate; but it is hoped rather that it may deal exclusively with a few well-established facts in such a manner, that the medical profession at large may be able to cope with the advertising physician in the treatment of the asthmatic.

In order to appreciate the logic of this paper, it will be necessary to briefly consider the blood in regard to its functions and also its chemico-physical and magnetic-electrical properties.

These functions and properties you are all familiar with, but those to be emphasised here are: (1) That it is a very delicate and sensitive substance of complex and varied functions; (2) That it is continually undergoing chemical changes, whose reactions are produced by the acid, alkaline and neutral fluids of the body, plus light, oxygen and the electricity of life; (3) That all foods that are assimilated, directly enter the blood, as chyle, and are immediately carried to the lungs for oxygenation.

Guided by these facts and schooled by experience, we can never fail to appreciate the value of a hygiene for the asthmatic. And the subjects that are of special interest and have immediate bearing upon this matter are, diet, habits, baths, light, altitude and location.

DIET.

No disease in the domain of medicine is more benefited by a proper diet, or aggravated by an improper one, than asthma. Foods entering the blood as they do almost immediately after ingestion, as chyle, do necessarily and almost directly generate and constitute it. This is beautifully illustrated by the vicious circle of asthma which is, indigestion, intestinal toxemia, impoverished blood, impaired oxygenation, asthma.

Occasionally the abnormal blood biochemy and its resultant impaired oxygenation, seem to precede the indigestion and intestinal toxemia, especially when due to the plasmodium malaria, but in most cases the vicious circle begins with the indigestion and intestinal toxemia.

In selecting a diet for the asthmatic, then, the crowning effort should ever be to choose such foods as are most rapidly assimilated and readily oxygenated. The food that has the greatest affinity for oxygen is blood, freshly drawn from the normal channels of a living animal. In fact, normal living blood's affinity for oxygen is so great that it is instantaneously altered by its presence, which renders the obtaining of it as a food for all asthmatics impractical, but it is in accordance with good logic to adopt as a diet its next of kin, which undoubtedly is fresh, rare beef-steak or roast.

Having an impoverished blood and knowing what constitutes its deficiency, it theoretically would be an easy matter to prescribe a food that would exactly counterbalance this deficiency, but practically it is a rather difficult undertaking, for we cannot alone take into consideration the deficiency of the blood and the foods containing this deficiency. Each case is a law unto itself and demands as a basic principle of treatment individualisation. No two patients are alike, either in the details of the disease, or in their customs, habits, constitutions, or chemistry of nutrition. Yet a rational diet for the asthmatic through our more accurate and complete knowledge of the nature of the affection, of digestion, nutrition, and of the various foods, is today possible.

In most asthmatic cases the chronic inanition is due to deficient alimentation, the diet being too exclusive or too reduced in quantity through fear or ignorance. After a meal rich in proteids the digestion leucocytosis, in the beginning of a course of enforced feeding, will increase the dyspnea after meals, but the blood is soon so altered and strengthened that the digestion leucocytosis is not noticed.

This digestion leucocytosis and its resultant increased dyspnea is the father of the popular, but decidedly erroneous idea that the asthmatic must starve. The blood being already so greatly impoverished that it cannot absorb or carry sufficient oxygen is temporarily rendered worse, diluted and more work thrown upon it by the addition of modified foods or chyle. Therefore the rational and only way to fan this impoverished blood back to its normal standard is by the graduated and frequent ingestion of foods rich in albumin and blood elements, as meats. I claim for a fresh, rare beefsteak or roast diet with fresh fruits, that it not only directly generates a blood favorable to oxygenation, but also one that is unfavorable to the osmotic outpouring of mucus, so characteristic in the asthmatic.

There is some connection between eosinophilia and asthma, but what and how have never been explained. But a point in favor of enforced proteid feeding of the asthmatic lies in the fact that the polymorphonuclear cells and eosinophiles are increased in chronic starvation and that the eosinophiles are diminished during digestion. There is also some connection between leukemia, leucocytosis and asthma, but the fact that herbivorous animals, and presumably vegetarians are exempt from the physiological leucocytosis of digestion is no more of an argument against the proteid diet for the asthmatic than it would be to argue that a boiler running with deficient steam should not have this deficiency increased by the abstraction of heat following the addition of fresh fuel. So also as fuel added to a low fire produces more smoke and less heat than when added to a hot one, does food call forth a greater leucocytosis in proportion to its novelty in the stomach. It is advisable, then, to change the accustomed diet, though it be improper, slowly and tentatively. Changes, and great changes, may be necessary, but they should be gradual and proportionate to the patient's power of adaptation.

It is my custom to begin enforced feeding through the progressive diet constructed by Leube, which is as follows: (1) Bouillon, Leube-Rosenthal meat solution; milk; soft-boiled or raw eggs; dry toast or crackers; water or neutral indifferent effervescent ($Co_2$) water. (2) Boiled calf's brains; boiled sweet-bread; (thymus of calf) boiled young chicken; boiled squab cereal soups; tapioca cooked in milk; boiled calfs' feet. (3) Sirloin pulp steak; grilled sirloin steak; scraped raw ham; mashed potatoes, baked with a little milk and butter; a little white bread; cup of hot water with milk and salt. (4) Roast beef; roast chicken; venison; partridge and veal; boiled lean fish; macaroni; bouillon or rice soup; spinach; a little wine. (5) Baked apple; all common foods; finally, salads, vegetables and stewed fruits.

Clinically, if not scientifically, the correctness of this diet rapidly proved itself, in that it is soon borne with ease, comfort and increased health by old asthmatics who have for years through fear of digestion, dyspnea and flatulency actually fasted. The hot water, salted with a teaspoonful of milk, furnishes an excellent substitute for tea and coffee. It supplies the necessary heat to stimulate digestion and the salt acts as a powerful and natural intestinal antiseptic.

The efficiency of sodium chloride, common table salt, in the prevention of putrefaction and decomposition is most strikingly demonstrated through the common practice of preserving animal and vegetable matter by salting. It is well ever to encourage the free use of fruits, rich in organic acids, such as lemons, sour cherries, vinegar pickles, sour apples, sour oranges and grapes. These acid fruits have a double action. They preserve the normal chemistry of the fluids of the body and also the equilibrum of digestion through their laxative properties. The value of fresh acid fruits in the maintenance of a normal blood is often and forcibly demonstrated by the occurrence of scurvy in cases where they cannot be obtained, as on board of ships and in prisons. Foods rich in alkalies must also be given freely, not so much for their solvent effect on uric acid as has heretofore been taught, but because alkalies and the normal alkalinity of the blood constitutes the basic principle of its oxygenation. The diet of the asthmatic should be varied and abundant. He should make eating his chief aim in life. Eat slowly, frequently, much, and a variety.

### BATHS.

Hydrotherapy ranks next in importance to diet. In fact it is safe to base the prognosis of an asthmatic case on the probable efficiency of its hydrotherapy. In cases that cannot for any reason, as fear, dread, ignorance or physical peculiarities avail themselves of these hydriatic procedures, the prognosis is grave. While in intelligent cases that are thoroughly convinced of the importance of hydrotherapy and the prospects are that it will be practised as ordered, the prognosis is good. There is no organ, tissue or substance in the body of an asthmatic that is not greatly and directly benefited by a proper system of cold water baths. The blood, though, is the substance most altered by cold water baths, and it is through this altered blood that the asthmatic derives the most of his benefit. Cold water baths and altitude exert a similar influence over the blood. That the curative effect of cold water sponges is due to an alteration of the blood has often been proven.

Knëpfelmacher has found immediately following cold water baths, given from ten to twelve minutes at a temperature of sixty-two to seventy-five degrees, that the blood cells rapidly increased in number. The increase began two minutes after the bath was entered. The maximum increase was 30 per cent. A diminution ensued in half an hour. The hemoglobin and specific gravity of the blood were in agreement with this result. Theoretically this increase in the cellular elements of the blood, which are the carriers of oxygen and carbonic oxide should have a decided therapeutic effect upon the dyspnea, and clinically the correctness of this theory is most forcibly demonstrated by the fact that an asthmatic may be suffering with agonising dyspnea that will disappear like magic in a bath tub of cold water. How cold water baths increase the cellular elements of the blood and thus its oxygen-carrying power is at present a disputed question, but that the blood cells are increased by cold water baths is conceded by all.

Winternitz claims that the effects are due to changes produced in the circulation through the increased action of the heart, tone of the vessels and tissues which drive the blood cells from organs in which stasis and accumulation of red and white blood cells have occurred. There have been many theories advanced to explain this increase in number of the corpuscular elements under the influence of cold, but the most rational explanation seems to be that offered by Winternitz, for this increase in the blood cells following cold water baths is too immediate to be due to the generation of new cells. Cold water baths improve the oxygen absorbing and carrying properties of the blood, not alone by the increase in the blood corpuscles, but also by beneficial changes in its chemical constituents.

Strasser has shown that after cold procedures the relative quantity of acid phosphate was diminished, and the alkalinity of the blood increased. Thus daily cold water baths will do much to create and maintain a normal blood, both as regards its alkalinity and corpuscular count, either of which is absolutely necessary for its perfect oxygenation.

A great deal of tact is necessary in prescribing this treatment for the asthmatic, as he has an inborn dread and fear of cold water, and the physician who simply tells his asthmatic patient to take a cold water bath once a day will probably never know the results of his prescribed treatment. It is my custom to begin with salt rubs. I have the patient take two quarts of hot water and make a saturated salt solution, using coarse barrel salt, then soak in this salt solution ten linen towels, about two and one-half feet long. After the towels are thoroughly saturated, hang, without wringing, and let them drain dry. This produces a rough salt towel with which I order the patient to rub himself for five minutes every morning for ten mornings. I then, in all allowable cases, order the cold water sponges.

It is necessary here to give very complete and careful directions to your patient. Patients should be instructed to obtain a large bathing sponge and half a dozen coarse Turkish towels. Then every morning on getting out of bed, they should run in the bath tub about four inches of warm water, at a temperature of about ninety degrees, stand in this and put the sponge in a large pail, run the pail full of cold water, or water at a temperature of about fifty-five, set the pail down in the tub or some convenient place, grasp the sponge and clasp it to the chest, letting cold water run down over abdomen, soak the sponge again and clasp it to the back, letting water run down the spine; sponge the arms and legs; jump out of the tub, take one towel and rub the water off the body, then take another dry towel and rub briskly for five minutes. These friction rubs, following the cold water, produce a delightful reaction and the patient will not need much urging to continue the process. After a few mornings the patient will usually voluntarily discontinue the hot water in bottom of tub and take a clear cold water sponge, in the manner above described.

### LIGHT.

Fuller has discovered remarkable changes produced by light upon the composition of the blood. What these changes are I do not know, but I am thoroughly convinced that sunlight has a most decided effect over the blood of the asthmatic. I believe that it is for the want of the chemical influence of sunlight that most asthmatics have their attacks during the night. The power of sunlight as a chemical agent is beautifully illustrated in photography and also in the chlorophly of plants growing without its influence. The chemical effect of sunlight on the blood is shown clinically by the fact that an asthmatic may suffer during the night with agonising dyspnea that immediately disappears in the morning upon getting a sun bath. The absence of the effect of sunlight on the blood of the asthmatic often proves to be the last straw in the production of an attack. Sunlight can be advantageously utilised in the hygiene of the asthmatic by choosing for him a sunny room in a sunny country.

The friction rubs and baths before mentioned should be taken in a room well lighted by day or sunlight and when possible under the direct solar rays. By thus taking these baths and friction rubs under the direct or indirect influence of the solar rays, we expose the entire surface of the body to light's action, and as a result obtain nearly double the alterative effect on the blood that would be obtained were these baths and rubs taken at night or in a dark room, as a poorly lighted basement. During these light exposures the temperature should not exceed sixty or seventy degrees, as excessive heat is debilitating to the blood. For this reason it is well to choose the morning hours for these combined cold water friction rubs and light baths.

ALTITUDE.

It has long been known that high altitudes exerted over the asthmatic a favorable influence, but how it did this was never until recently discovered. There remains no doubt now, however, but that the benefit is directly due to the immediate increase in the number of red blood corpuscles. This polycythemia increases the higher one goes.

Vialt has found an increase from 4,974,000 red cells at sea level to 8,000,000 at an altitude of 14,224 feet. Old asthmatics who have long resided at or about the sea level, and though they may have had asthma in its severest forms for years there, are on ascending to high altitudes, as in the mountains of Colorado, instantaneously relieved and temporarily cured. It is not practical to utilise the beneficial influence of altitude as a hygienic principle in the treatment of all asthmatics, but it can be advantageously used in the treatment of those who might for any reason prefer it to drugs. Asthmatics, though, who rely on altitude alone as a cure, usually fail. The cure is successful enough while they continue to live at a high altitude, but on resuming their former sea level residence, their asthma is quite apt to return. This returning of asthma at sea level can, however, in time and by the observance of proper hygienic measures, be in most cases, prevented, and the altitude cure made effectual and permanent, thus robbing this cure of a popular prejudice that all those who avail themselves of it must become exiles from home. The requisite hygienic reform should begin during the altitude sojourn and be continued at the sea level abode.

HABITS.

There is no class of people more prone to habit than the asthmatic. Physicians not knowing the nature of the disease have tried one drug after another, until his victim either died, deserted or accidentally became cured. If the latter, the physician would declare the drug he was using when this accident occurred, to be a specific, and all of his asthmatic patients were put on this supposed specific and continued thereon till they had a drug habit. The habit affliction, though, is not alone imposed by the physician, for each and every grandparent, uncle, aunt and friend of the asthmatic have a sure cure that must be faithfully tried.

What wonder, then, that the asthmatic becomes a bundle of habits that collectively or individually are his enemies. Tobacco and alcohol are the two habits that are most usually found associated with the asthmatic, and I believe they should be strictly interdicted. Alcohol, during severe asthmatic seizures can be advantageously used, but its habitual or accustomed use at other times should never be tolerated. Tobacco should never be allowed in any form or at any time. It is quite true that tobacco, when pushed to its physiological limits will in an asthmatic, when unaccustomed to its use, produce temporary relief, if given during a severe attack. But this relief is purchased by an extravagant expenditure of blood recuperating power of which the asthmatic has none to spare, and it is followed by such a debilitating condition as faintness, giddiness, nausea and vomiting, cardialgia, torpor, sleepiness, palpitation of the heart, hypochondriasis, amblyopia, general relaxation of the muscular system, trembling, complete prostration of strength, coldness of the surface, cold clammy perspiration and convulsive movements. These symptoms, one and all, have been experienced by every tobacco smoker.

Marable, in an excellent paper on tobacco, quotes Dudley, who has shown by experiments that when there is a layer of fire, one sixty-fourth to one sixteenth of an inch in thickness as the air is drawn through the hot carbon, this is reduced to carbon monoxide and as such is drawn into the mouth, for when it passes beyond the fire there is no air or oxygen to convert it back to carbon dioxide. Its well-known poisonous effects when inhaled are the results of its affinity for the hemoglobin of the blood, converting the oxyhemoglobin into the carbonic oxid hemoglobin, a stable compound not reduced in the circulation, hence producing permanent asphyxia. Gas, smoke and all the results of combustion are the end products of oxygenation, and are therefore directly poisonous to it, as are the end products of any of nature's phenomena directly poisonous to a second production of those phenomena.

In consideration of these simple facts it seems strange that the medical profession, with their superabundance of knowledge, should continue to wonder whether or not tobacco smoke is detrimental to mankind, and ever poison themselves and their patients with the end products of oxygenation, thus crippling one of life's most vital functions.

In those who have a healthy vigorous bood that rapidly absorbs and readily carries oxygen, the impaired oxygenation resulting from tobacco smoke will hardly be noticed, but with the asthmatic it is the last straw and must be strictly prohibited.

LOCATION.

I believe that most asthmatics can be cured in any country or clime by a proper use of drugs and hygienic procedures. But altitude and location may in a vast majority of cases be advantageously substituted for drugs. The main object, other than altitude, in choosing a location for an asthmatic is to avoid malarial districts, for there is no disease more destructive to the cellular constituents of the blood, or that better paves the way for asthmatic seizures than malaria. The asthmatic, then, must either avoid malarial districts or be compelled to occasionally resort to an anti-malarial treatment.

Many authorities have shown, without attempting an explanation, that the country bred asthmatic was greatly benefited, if not entirely cured, on assuming a city abode. This statement can now be followed by a rational explanation for the benefit the asthmatic derives from a city life is most decidedly due to the fact that our modern cities are, as a rule, exempt from malarial infections.

This completes the hygienic circle of the asthmatic and proves that circle theoretically, scientifically, practically and clinically to be diet, baths, habits, location, polycythemia and health.

